# Effect of the Hydroethanolic Extract of *Bixa orellana* Linn (Bixaceae) Leaves on Castor Oil-Induced Diarrhea in Swiss Albino Mice

**DOI:** 10.1155/2019/6963548

**Published:** 2019-12-01

**Authors:** Michel Archange Fokam Tagne, Hypolyte Akaou, Paul Aimé Noubissi, Angèle Foyet Fondjo, Yaouke Rékabi, Henri Wambe, René Kamgang, Jean-Louis Essame Oyono

**Affiliations:** ^1^Department of Biological Science, Faculty of Science, University of Ngaoundere, Cameroon; ^2^Department of Zoology and Animal Physiology, Faculty of Science, University of Buea, Cameroon; ^3^Department of Applied Sciences for Health, Higher Institute of Applied Sciences, University Institute of Gulf of Guinea, Cameroon; ^4^Department of Biological Science, Faculty of Science, University of Dschang, Cameroon; ^5^Department of Animal Biology and Physiology, Faculty of Science, University of Yaoundé I, Cameroon; ^6^Laboratory of Endocrinology and Radioisotopes, Institute of Medical Research and Medicinal Plants Studies (IMPM), Yaounde, Cameroon

## Abstract

**Objective:**

The treatment of diarrheal diseases is a serious problem in developing countries, where population generally uses medicinal plants. The leaves of *Bixa orellana* have been reported to be traditionally used in the treatment of diarrhea by local people in the district of Khulna in Bangladesh. The aim of this study was to investigate the effects of the hydroethanolic extract of *Bixa orellana* leaves on castor oil-induced diarrhea in mice.

**Methods:**

The powder of the leaves of *Bixa orellana* was macerated in ethanol/water mixture (20/80) for 48 hours and then filtered. The filtrate obtained was lyophilized, and the solutions to be administered to the animals were prepared. To induce diarrhea, animals orally received castor oil (1 mL/100 g bw). To determine the effective doses, each mouse received, 30 minutes after the administration of castor oil, one of the single oral doses of hydroethanolic extract of *Bixa orellana* leaves: 0, 25, 50, 100, and 200 mg/kg bw. The mass, number, and frequency of stool diarrhea were measured and recorded per hour for five hours. The effect of the hydroethanolic extract of *Bixa orellana* leaves on the intestinal transit was evaluated by measuring the distance traveled by the charcoal meal in thirty minutes. The effects of the aqueous extract of hydroethanolic extract of *Bixa orellana* leaves on intestinal secretion were evaluated by measuring the volume of the intestinal content and by dosing the electrolytes (Na^+^, K^+^, and Cl^−^) in the intestinal content by the colorimetric method.

**Results:**

The extract produced significant (*P* < 0.01) decreases, respectively, 35.52%, 54.47%, 74.80%, and 87.80% in the severity of diarrhea. The extract at 100 and 200 mg/kg bw showed a significant (*P* < 0.01) decrease of castor oil-induced enteropooling (61.08% and 65.41%), and only the 200 mg/kg bw exhibited significant (*P* < 0.01) reduction on intestinal transit (24.46%) as compared to standard drug.

**Conclusions:**

The hydroethanolic extract was found to be effective against castor oil-induced diarrhea in experimental mice at 50, 100, and 200 mg/kg bw which provides evidence that could justify its traditional use.

## 1. Introduction

In developing countries, diarrheal diseases are a major cause of suffering and millions of deaths every year. These diseases represent the most serious problem in these countries, affecting more children and are considered the second leading cause of death in children under five [1, 2]. Diarrhea most of the time refers to a disease and sometimes is a symptom of other disease conditions. It is associated with infectious agents (viral, bacterial, and fungus infection), food poisoning, and other disease conditions of the gastrointestinal disorder, characterized by an increase in stool frequency [2]. Diarrhea is incontinence and a fecal urgency associated with an imbalance between the mechanisms of intestinal absorption and secretion. This imbalance usually results in excessive loss of body fluids and electrolytes in the stool and is often accompanied by intestinal hypermotility [2]. The major categories of diarrhea most commonly encountered are secretory diarrhea with an osmotic gap < 50 mOsmol/L, osmotic diarrhea with an osmotic gap > 125 mOsmol/L, and motor diarrhea resulting from decreased digestive tract diameter, water malabsorption, and increased intestinal peristalsis [3]. The use of synthetic drugs such as diphenoxylate, atropine maleate, kaolin, pectin, antibiotics, and oral rehydration solutions in the treatment of diarrhea is often associated with many problems such as accessibility, high cost of drugs, and the multiresistance of microorganisms. These problems related to modern medicine are driving many people in developing countries to turn to local traditional medicine that uses medicinal plants in the treatment of these diarrheal diseases [4]. In the world, medicinal plants are a source of major compounds used as drugs or therapeutic agents [5] and many of which are effective. In many countries, medicinal plants are alternative sources of drugs for the majority of the population who do not have access to conventional medicine. The lack of less toxic synthetic drugs available to fight common conditions such as diarrhea has prompted many people to turn to herbal remedies that can relieve the disease or permanently control the secretory process leading to diarrhea [6].

Several medicinal plants have already scientifically shown their efficiencies with less harm in the treatment of diarrhea. We can mention among others, *Oxalis barrelieri* [7, 8], *Crinum jagus* [9, 10], *Anogeissus leiocarpus* [11], *Euphorbia scordifolia* [12, 13], and *Bixa orellana* [14]. *Bixa orellana* is widely used in traditional medicine for the prevention and treatment of a large number of diseases such as headaches, blood disorders, gonorrhea, fever, epilepsy, dysentery, and jaundice [15]. People in Bangladesh use the leaves of this plant to treat diarrhea, insomnia, and skin diseases [16]. The methanolic extract of *Bixa orellana* showed maximal antidiarrheal activity (85%) at 500 mg/kg [16]. In Cameroon, *Bixa orellana* is widely available in Ngaoundere (Adamawa region) and is traditionally used (maceration of the leaves in the white wine of raffia or decoction) by this population in the treatment of joint pain, jaundice, fever, and abdominal pain. Given that the effectiveness of a plant extract depends on the location, the season, and the harvesting time off, as well as the mode of extraction of the plant, the main objective of this work was to evaluate the antidiarrheal properties of the hydroethanolic extract of *Bixa orellana* leaves to prove its effectiveness in the treatment of diarrheal diseases in Cameroon.

## 2. Materials and Methods

### 2.1. Plant Material

The fresh leaves of *Bixa orellana* were harvested at the Ngaoundere (Adamawa region of Cameroon) University campus in July 2018 between 8 and 10 am. The plant was then identified at the Cameroon National Herbarium in comparison with the material of Surville N. No. 349 of the specimen in the Herbier collection No. 14099/SRF.Cam.

The leaves of *Bixa orellana* were harvested and thoroughly washed in water to remove dirt. These leaves were dried in the shade at room temperature and then ground to a dried powder. Two hundred grams (200 g) of this powder was macerated in 2 L of ethanol/water mixture (20/80) for 72 hours. After the maceration period, the macerated powder was then pressed and the residue was remacerated. Plant extract solutions obtained were then filtered using the Whatman No.1 filter paper. The filtrate was aliquoted in bottles, frozen at -35°C, and then lyophilized with a freeze dryer (CHRIST, ALPHA 1-2.LD.plus) to obtain 27.50 g of crude extract, a yield of 13.75%. Prior to oral administration, the extract was dissolved in distilled water so that each animal received no more than 10 mL/kg of body weight.

### 2.2. Preliminary Phytochemical Screening Test

The qualitative phytochemical screening of the hydroethanolic extract of *Bixa orellana* was performed using the following chemical reagents and tests [17–19]: Dragendorff and Mayer reagents for the determination of alkaloids, ferric chloride (FeCl_3_) and K_3_Fe[(CN)_6_] for tannins, Liebermann-Burchard test for terpenoid and sterol, Shinoda test for flavonoids, frothing test for saponins, Molisch test for polysaccharides, UV lamps for coumarins, and Borntrager's test for anthraquinones.

### 2.3. Experimental Animals

Young, healthy Swiss Albino mice, 12 weeks old, weighing between 20 and 25 g, of either sex, obtained from the animal house of the Faculty of Sciences of the University of Ngaoundere, Cameroon, were used for this work. Animal housing and *in vivo* experiments were done according to the guidelines of the European Union on Animal Care (CEE Council 86/609) [20] that were adopted in Cameroon by the Institutional Committee of the Ministry of Scientific Research and Innovation. The mice were housed in polyethylene cages (5 animals per cage) and acclimated for one week under the environmental conditions of the laboratory prior to the study. During the acclimation period, the animals had free access to food and water.

### 2.4. Activity of the Hydroethanolic Extract of *Bixa orellana* Leaves on Castor Oil-Induced Diarrhea in Mice

Six groups of five mice each were fasted for 18 h with free access to water. To induce diarrhea, castor oil (0.5 mL) was orally administered to all mice [21]. Thirty minutes after castor oil administration, the first group (diarrheal control: DC) received distilled water (10 mL/kg bw), the second group received the reference drug, Loperamide (ELDOPER, Micro Labs, 92, sipcot, Hosur-635126, India) 5 mg/kg bw, while the other four (4) groups received one of the *Bixa orellana* hydroethanolic extract doses: 25, 50, 100, or 200 mg/kg bw by oral route. After these treatments, each mouse was individually placed in a metabolic cage lined with previously weighed filter paper. These filter papers were changed every hour for 5 h [22]. During each hour, total stool mass, diarrhea stool mass, total stool count, and the number of diarrheal stool wet feces were recorded. The percentage inhibition of diarrhea and the stool emission frequency (SEF) were calculated as follows [11]:

(1) $$\begin{array}{c} \text{SEF}=\frac{\text{Total number of stool}}{\text{Time }\left(5\text{ h}\right)}, \end{array}$$

(2) $$\begin{array}{c} I\;\left(\%\right)=\frac{\text{SMDC}-\text{SMDT}}{\text{SMDC}}*100, \end{array}$$

where *I* is the inhibition; SMDC is the stool mass of diarrheal control; SMDT is the stool mass of diarrheal test.

### 2.5. Activity of the Hydroethanolic Extract of *Bixa orellana* Leaves on Intestinal Transit in Mice

Five groups of five normal mice each were fasted for 18 h with free access to water. The normal control group (NC) received distilled water (10 mL/kg bw). The second group (AT0.3) received the standard drug: atropine sulfate (Gland Pharma, Pally, Dundigal Post, Hyderabad, India) 0.3 mg/kg bw i.p. The three other groups received one of the *Bixa orellana* leaf hydroethanolic extract doses: 50 mg/kg (HEBo50), 100 mg/kg (HEBo100), and 200 mg/kg (HEBo200). Thirty minutes after these treatments, each mouse received 1 mL of charcoal meal (5% activated charcoal by 5% gum acacia), and 30 minutes later, the animals were sacrificed by cervical dislocation. The abdomen of each animal was opened, and the intestine was removed. The total distance of the small intestine and the distance traveled by the charcoal meal in the intestine were measured with a tape measure and then expressed as a percentage of the total distance traveled from the pylorus to the caecum [23]. The peristaltic index (PI) was calculated as follows [11]:

(3) $$\begin{array}{c} \text{PI}=\frac{\text{CCL}}{\text{ITL}}*100, \end{array}$$

where PI is the peristaltic index, CCL is the charcoal covered length, and ITL is the intestine total length.

### 2.6. Activity of the Hydroethanolic Extract of *Bixa orellana* Leaves on Castor Oil-Induced Enteropooling in Mice

Six groups of five mice each were fasted for 18 h with free access to water. The normal control (NC) group received distilled water (10 mL/kg bw). The other five groups received castor oil (0.5 mL). Thirty minutes after castor oil administration, the diarrhea control (DC) group received distilled water (10 mL/kg bw), the third group (Lop5) received the reference drug, Loperamide (ELDOPER, Micro Labs, 92, sipcot, Hosur-635126, India) 5 mg/kg bw, while the other three (03) groups received one of the *Bixa orellana* hydroethanolic extract doses: 50 mg/kg bw (HEBo50), 100 mg/kg bw (HEBo100), or 200 mg/kg bw (HEBo200) by oral route. One hour later, the mice were sacrificed, the small intestine removed and weighed with its content. The contents of the small intestine were further emptied into a graduated tube, its volume measured and the emptied small intestine reweighed [23, 24]. The intestinal content was used for the determination of electrolytes (sodium, potassium, and chloride) levels.

### 2.7. Electrolyte Assessment and Stool Osmotic Gap

#### 2.7.1. Determination of Chloride Ions

The chloride ions were assayed by the colorimetric method using the kit (SGM Italia-Via Pindaro 28C-Roma). The chloride ions react with mercury thiocyanate-releasing thiocyanate ions which form with trivalent iron a colored compound whose intensity of color is proportional to the chloride concentration.

One thousand microliters (1000 *μ*L) of the reagent was put in all the tubes. We then added 10 *μ*L of distilled water in the white tube, 10 *μ*L of standard solution (100 mEq/L) in the standard tube, and 10 *μ*L of samples in the test tubes. Each mixture was incubated for five (5) minutes at 37°C in a water bath, and then, the optical densities (OD) were read with the spectrophotometer (UNICO© ULTRA VIOLET) at 480 nm. The concentration of the chloride ions in each tube was determined by the following formula:

(4) $$\begin{array}{c} \left[{\text{Cl}}^{-}\right]\;\left(\text{mEq}/\text{L}\right)=\frac{\text{Abs T}}{\text{Abs S}}*\left[\text{S}\right], \end{array}$$

where [Cl^−^] is the concentration of chloride ions in the intestine content; Abs T is the absorbance of the test tube; Abs S is the absorbance of the standard tube; [S] is the standard concentration of chloride ions (100 mEq/L).

#### 2.7.2. Determination of Sodium Ions

The sodium ions were assayed by colorimetric method using the kit (LIQUIZYME SODIUM, BEACON DIAGNOSTICS PVT. LTD., 424 New GIDC, Kabilpore, Navsari-396 424, India). Sodium ions react with the selective chromogen and produce a chromophore whose absorbance is directly proportional to the concentration of sodium in the sample.

Five hundred microliters (500 *μ*L) of the reagent was put in all the tubes. We added 10 *μ*L of distilled water in the blank tube, 10 *μ*L of standard solution (150 mEq/L) in the standard tube, and 10 *μ*L of samples in the test tubes. Each mixture was incubated in a water bath for five (5) minutes at 37°C, and then, the absorbance was read at 630 nm with a spectrophotometer (UNICO© ULTRA VIOLET). The concentration of sodium ions in each tube was determined by the following formula:

(5) $$\begin{array}{c} \left[\text{N}{\text{a}}^{+}\right]\;\left(\frac{\text{mEq}}{\text{L}}\right)=\frac{\text{Abs T}}{\text{Abs S}}*\left[\text{S}\right], \end{array}$$

where [Na^+^] is the concentration of sodium ions in the intestine content; Abs T is the absorbance of the test tube; Abs S is the absorbance of the standard tube; [S] is the standard concentration of chloride ions (150 mEq/L).

#### 2.7.3. Determination of Potassium Ions

The potassium ions were assayed by colorimetric method using the kit (LIQUIZYME POTASSIUM, BEACON DIAGNOSTICS PVT. LTD., 424 New GIDC, Kabilpore, Navsari-396 424, India). The potassium ion reacts with tetraphenol in a specially prepared buffer to form a colloidal suspension. The amount of turbidity produced is directly proportional to the potassium concentration in the sample.

Five hundred microliters (500 *μ*L) of the reagent was put in all the tubes. We then added 20 *μ*L of distilled water in the white tube, 20 *μ*L of standard solution in the standard tube, and 20 *μ*L of samples in the test tubes. Each mixture was incubated in a water bath for five (5) minutes at 37°C, and the absorbance of the contents of each tube was read at 630 nm with the spectrophotometer (UNICO ULTRA VIOLET). The concentration of potassium ions in each tube was determined by the following formula:

(6) $$\begin{array}{c} \left[{\text{K}}^{+}\right]\;\left(\frac{\text{mEq}}{\text{L}}\right)=\frac{\text{AbsT}}{\text{AbsS}}*\left[\text{S}\right], \end{array}$$

where [K^+^] is the concentration of potassium ions in the intestine content; Abs T is the absorbance of the test tube; Abs S is the absorbance of the standard tube; [S] is the standard concentration of chloride ions (5 mEq/L).

#### 2.7.4. Determination of Stool Osmotic Gap (SOG)

The stool osmotic gap was calculated according to the formula [25]

(7) $$\begin{array}{c} \text{OG}=290-2*\left(\left[\text{N}{\text{a}}^{+}\right]+\left[{\text{K}}^{+}\right]\right), \end{array}$$

where [Na^+^] is the concentration of sodium ions in the intestine content, and [K^+^] is the concentration of potassium ions in the intestine content.

### 2.8. Statistical Analysis

Data were expressed as mean ± standard error of mean (*X* ± S.E.M). Data were analyzed by one-way ANOVA followed by Dunnett's *t*-test using computerized GraphPad InStat (DATASET1.ISD). Values were considered significant at *P* < 0.05 in comparison with the control.

## 3. Results

### 3.1. Phytochemical Compounds of the Hydroethanolic Extract of *Bixa orellana* Leaves

The phytochemical screening of the hydroethanolic extract of *Bixa orellana* revealed the presence of terpenes, flavonoids, tannins, coumarins, and saponins. However, the absence of alkaloids and anthraquinones was noted (Table 1).

### 3.2. Antidiarrheal Activity of Hydroethanolic Extract of *Bixa orellana* Leaves

A few minutes after castor oil administration, mice became less mobile and folded on themselves with erect hairs. The first diarrheal stools appeared generally on the first and rarely at the second hour in the treated mice. Five (5) hours after the administration of castor oil, diarrheal controls emitted 0.25 ± 0.02 g and 3.00 ± 0.71 N/h of diarrheal stools. These diarrheal stools significantly (*P* < 0.05) decreased in animals treated with the hydroethanolic extract of *Bixa orellana* leaves at 50 mg/kg (HEBo50), 100 mg/kg (HEBo100), and 200 mg/kg (HEBo200). The inhibition rate of diarrhea was 84.00%, 32.00%, 56.00%, 76.00%, and 88.00%, respectively, in mice treated with Loperamide 5 mg/kg bw (Lop5), *Bixa orellana* leaf hydroethanolic extract at 25 mg/kg (HEBo25), 50 mg/kg (HEBo50), 100 mg/kg (HEBo100), and 200 mg/kg (HEBo200). The frequency of stool emission was, respectively, 3.0, 0.6, 1.8, 0.8, 0.6, and 0.2/h for diarrheal control, Lop5, HEBo25, HEBo50, HEBo100, and HEBo200 (Table 2).

### 3.3. Effect of the Hydroethanolic Extract of *Bixa orellana* Leaves on Intestinal Transit

The progress of charcoal meal in the gastrointestinal tract was 76.45 ± 5.01%, 48.13 ± 1.71%, 55.23 ± 2.28%, 50.81 ± 0.89%, and 47.45 ± 2.68%, respectively, in the normal control (NC), the group treated with atropine sulfate 0.3 mg/kg (AT0.3), and the groups treated with the *Bixa orellana* leaf hydroethanolic extract at 50 mg/kg (HEBo50), 100 mg/kg (HEBo100), and 200 mg/kg (HEBo200). The inhibition of intestinal transit was 37.04%, 27.75%, 33.54%, and 37.94%, respectively (Table 3).

### 3.4. Effect of the Hydroethanolic Extract of *Bixa orellana* Leaves on Castor Oil-Induced Enteropooling

The difference in mass between the untrimmed bowel and the emptied bowel was 0.37 ± 0.13 g, 0.21 ± 0.01 g, 0.26 ± 0.06 g, 0.33 ± 0.04 g, 0.28 ± 0.07 g, and 0.24 ± 0.09 g, respectively, in the diarrheal control (DC), the normal control (NC), the treated group with Loperamide 5 mg/kg (Lop5), and the test groups treated with *Bixa orellana* leaf hydroethanolic extract at 50 mg/kg (HEBo50), 100 mg/kg (HEBo100), and 200 mg/kg (HEBo200) (Table 4).

**Figure 1:**
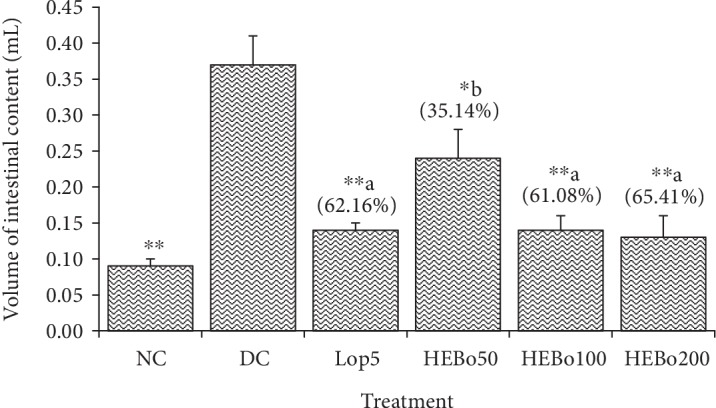
Effect of the hydroethanolic extract of *Bixa orellana* leaves on the intestinal secretion. Data are expressed as mean ± S.E.M (*n* = 5). NC: normal control; DC: diarrheal control; Lop5: Loperamide 5 mg/kg; HEBo50, HEBo100, and HEBo200: hydroethanolic extract of *Bixa orellana* leaves, respectively, at 25, 50, 100, and 200 mg/kg; (%): inhibition. Significant difference: ^∗^*P* < 0.05 and ^∗∗^*P* < 0.01 compared with DC; ^a^*P* < 0.05 and ^b^*P* < 0.01 compared with NC.

The volume of intestinal contents was 0.09 ± 0.01 mL, 0.37 ± 0.04 mL, 0.14 ± 0.01 mL, 0.24 ± 0.04 mL, 0.14 ± 0.02 mL, and 0.13 ± 0.03 mL, respectively, in the normal control (NC), the diarrheal control (DC), the Loperamide-treated group, 5 mg/kg (Lop5), and the test groups treated with *Bixa orellana* hydroethanolic extract at 50 mg/kg (HEBo50), 100 mg/kg (HEBo100), and 200 mg/kg (HEBo200). The inhibition of secretion was 35.14%, 61.08%, 65.41%, and 62.16%, respectively (Figure 1).

### 3.5. Effect of the Hydroethanolic Extract of *Bixa orellana* Leaves on Electrolyte Concentration and Stool Osmotic Gap

Concentrations of chloride ions were 126.39 ± 4.18 mEq/L in diarrheal controls and 97.59 ± 7.08 mEq/L in normal control. Compared to diarrheal controls, these values significantly decreased (*P* < 0.01) in treated animals and were 91.27 ± 8.17 mEq/L, 103.18 ± 6.14 mEq/L, 108.61 ± 5.12 mEq/L, and 106.39 ± 3.08 mEq/L, respectively, for animals treated with Loperamide or with *Bixa orellana* leaf hydroethanolic extract at 50 mg/kg, 100 mg/kg, and 200 mg/kg. Sodium ion concentrations in animals treated with *Bixa orellana* leaf hydroethanolic extract or Loperamide were significantly (*P* < 0.01) low compared to diarrhea control (129.60 ± 8.24 mEq/L) and were, respectively, 107.82 ± 4.46 mEq/L, 106.91 ± 6.47 mEq/L, and 106.33 ± 4.85 mEq/L at 50, 100, and 200 mg/kg or 103.16 ± 3.82 mEq/L for Loperamide 5 mg/kg. Potassium ion concentrations were 10.51 ± 2.74 mEq/L, 5.75 ± 1.01 mEq/L, 11.05 ± 3.41 mEq/L, 9.89 ± 0.68 mEq/L, 10.54 ± 0.43 mEq/L, and 10.02 ± 2.91 mEq/L, respectively, in normal control, diarrheal control, and in animals treated with Loperamide or with hydroethanolic extract of *Bixa orellana* leaves at 50 mg/kg, 100 mg/kg, and 200 mg/kg (Table 5).

**Figure 2:**
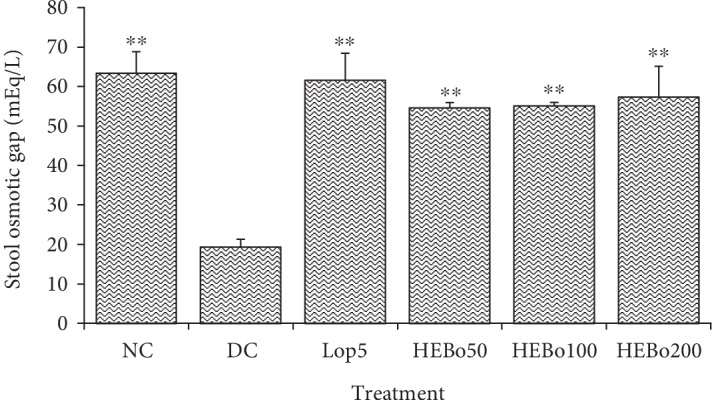
Effect of the hydroethanolic extract of *Bixa orellana* leaves on stool osmotic gap. Data are expressed as mean ± S.E.M (*n* = 5). NC: normal control; DC: diarrheal control; Lop5: Loperamide 5 mg/kg; HEBo25, HEBo50, HEBo100, and HEBo200: hydroethanolic extract of *Bixa orellana* leaves, respectively, at 25, 50, 100, and 200 mg/kg. Significant difference: ^∗∗^*P* < 0.01 compared with DC.

In diarrheal control, stool osmotic gap was less than 50 mEq/L and was 19.30 mEq/L. In all other animals, the stool osmotic gap was greater than 50 mEq/L and was 63.30 mEq/L, 61.58 mEq/L, 54.58 mEq/L, 55.10 mEq/L, and 57.30 mEq/L, respectively, in normal control and in animals treated with Loperamide or with *Bixa orellana* leaf hydroethanolic extract at 50, 100, and 200 mg/kg (Figure 2).

## 4. Discussion

The leaves of *Bixa orellana* have been reported to be traditionally used in the treatment of diarrhea by local people in the district of Khulna in Bangladesh, and scientifically, the methanolic extract of its leaves showed to have a maximum antidiarrheal activity at 500 mg/kg [16]. In this study, the hydroethanolic extract of leaves of *B. orellana* was prepared to investigate its effects on castor oil-induced diarrhea.

Depending on its etiology, diarrhea may be characterized by increased secretion of electrolytes (secretory diarrhea), increased luminal osmolality (osmotic diarrhea), decreased electrolyte absorption, and/or increased intestinal motility responsible for reduced transit time. Many antidiarrheal agents can therefore act by increasing the transit time by inhibiting gastrointestinal motility, inhibiting intestinal secretions, and/or increasing the intestinal absorption of water and electrolytes. Laxatives such as castor oil induce diarrhea by increased motility and/or gastrointestinal secretions via its active ingredient, the ricinoleic acid. Ricinoleic acid modifies the permeability of the intestinal mucosa to electrolytes [26] by inhibiting the intestinal Na^+^/K^+^ ATPase activity [8] and stimulating the biosynthesis and release of the endogenous prostaglandins responsible for diarrhea [27]. In this study, the hydroethanolic extract of *Bixa orellana* leaves at 200 mg/kg bw significantly and maximumly (88%) reduced the amount and frequency of castor oil-induced diarrheal stools in mice, unlike the methanolic extract which showed maximal inhibition (85%) at 500 mg/kg. Our extract could act by inhibiting one or more of the mechanisms of induction of diarrhea by castor oil.

*Bixa orellana* extract (200 mg/kg bw) significantly inhibited intestinal peristalsis almost as the reference drug, atropine. Atropine is a competitive antagonist of acetylcholine muscarinic receptor [28] located on the intestinal smooth muscle. The activation of these receptors induces the production of the second messengers IP_3_ and Diacyl glycerol (DAG) which are responsible for the increase of cytosolic calcium and smooth muscle contraction [11, 29, 30]. Like in the ethanolic extract of *Bixa Orellana* [14], phytochemical studies of the hydroethanolic extract revealed the presence of flavonoids, triterpenes, and tannins. These molecules would act either by blocking muscarinic receptors or by activating the opioid *μ* receptors located on the smooth intestinal muscle and thus reducing intestinal peristalsis [31–35].

Castor oil induced secretory diarrhea in mice because the osmotic gap was less than 50 mEq/L [25, 36]. The hydroethanolic extract of *Bixa orellana* leaves at 100 and 200 mg/kg bw reduced the volume and mass of the intestinal contents and maintained normal the value of the stool osmotic gap (greater than 50 mEq/L) almost like the reference drug, Loperamide. The antidiarrheal action of Loperamide can be mediated by an antisecretory mechanism. Loperamide is an opioid that affects intestinal absorption and inhibits its secretion by acting on the *δ* receptor. The antisecretory effect of Loperamide can be explained by the inhibition of the mechanisms leading to the synthesis of cAMP and cGMP, by the activation of Na^+^/K^+^ ATPase [37] or by stimulating cotransport of sodium chloride from the intestine and inhibiting calmodulin [25, 36]. In the presence of Mg^2+^ ions, the Na^+^/K^+^ ATPase catalyzes the release of Na^+^ and the entry of two K^+^ ions. The Na^+^/K^+^ ATPase is expressed in all intestinal epithelial cells and acts to maintain a low intracellular concentration of Na^+^ and a high intracellular concentration of K^+^ [38]. The inactivation of the Na^+^/K^+^ ATPase pump with ricinoleic acid inhibits all these active transport mechanisms in the intestine. This could indicate that the effect of the hydroethanolic extract of *Bixa orellana* leaves could be acted by an antisecretory mechanism similar to that of Loperamide by inhibiting the synthesis of cAMP and cGMP or by activating the Na^+^/K^+^ ATPase activity. These results could be explained by the presence in our hydroethanolic extract of antidiarrheal compounds such as flavonoids, tannins, terpenes, saponins, and sterols which are known for their antidiarrheal activities either as a transit inhibitor or as an antisecretory agent [31–34]. Indeed, it has been shown that flavonoids act through *α*_2_-adrenergic and calcium systems to exert their inhibitory effect on intestinal functions [31].

## 5. Conclusion

The hydroethanolic extract of *Bixa orellana* leaves inhibited diarrhea, reduced stool frequency, and inhibited intestinal peristalsis as well as water and electrolyte secretions. These effects prove the antidiarrheal activity of the hydroethanolic extract of *Bixa orellana* leaves and would justify the use of this plant in the treatment of diarrheal diseases. The precise mechanisms of this hydroethanolic extract of *Bixa orellana* leaves, which are responsible for the antidiarrheal activity, would be clarified by further studies.

## Figures and Tables

**Table 1:** Phytochemical compounds of the hydroethanolic extract of *Bixa orellana* leaves.

Class of compound	Reaction
Terpenes	+
Flavonoids	+
Tannins	+
Coumarins	+
Saponins	+
Polysaccharides	−
Anthraquinones	−
Alkaloids	−

+: present; −: absent.

**Table 2:** Effect of the hydroethanolic extract of *Bixa orellana* leaves on stool mass and stool frequencies in mice.

Group	Treatment	Total feces (g)	Diarrheal feces (g)	Inhibition (%)	Frequency (number/h)
DC	H_2_O (10 mL/kg)	0.28 ± 0.02	0.25 ± 0.02	—	3.00 ± 0.71
Lop5	5 mg/kg	0.06±0.02^∗∗^	0.04±0.04^∗∗^	84.00	0.60 ± 0.60^∗^
HEBo25	25 mg/kg	0.20 ± 0.02^b^	0.17 ± 0.03^b^	32.00	1.80 ± 0.49^b^
HEBo50	50 mg/kg	0.15 ± 0.09^∗^^b^	0.11 ± 0.09^∗^^b^	56.00	0.80 ± 0.49^∗^
HEBo100	100 mg/kg	0.10±0.04^∗∗^	0.06±0.04^∗∗^	76.00	0.60 ± 0.24^∗^
HEBo200	200 mg/kg	0.04±0.03^∗∗^	0.03±0.03^∗∗^	88.00	0.20±0.20^∗∗^

Values are expressed as mean ± S.E.M (*n* = 5). DC: diarrheal control; Lop5: Loperamide 5 mg/kg; HEBo25, HEBo50, HEBo100, and HEBo200: hydroethanolic extract of *Bixa orellana* leaves, respectively, at 25, 50, 100, and 200 mg/kg. Significant difference: ^∗^*P* < 0.05; ^∗∗^*P* < 0.01 compared with DC; ^b^*P* < 0.05 compared with Lop5.

**Table 3:** Effect of *Bixa orellana* leaf hydroethanolic extract on intestinal transit in mice.

Treatment	ITL (cm)	CCL (cm)	PI (%)	Inhibition (%)
NC	51.80 ± 1.50	39.60 ± 3.00	76.45 ± 5.01	—
AT0.3	48.20 ± 0.60	23.20 ± 1.10	48.13±1.71^∗∗^	37.04
HEBo50	38.20 ± 0.60	21.10 ± 1.10	55.23±2.28^∗∗^	27.75
HEBo100	49.40 ± 1.60	25.10 ± 3.00	50.81±0.89^∗∗^	33.54
HEBo200	47.00 ± 1.30	22.30 ± 3.10	47.45±2.68^∗∗^	37.94

Values are expressed as mean ± S.E.M (*n* = 5). ITL: intestine total length; CCL: charcoal covered length; PI: peristaltic index; NC: normal control; AT0.3: atropine sulfate 0.3 mg/kg; HEBo25, HEBo50, HEBo100, and HEBo200: hydroethanolic extract of *Bixa orellana* leaves, respectively, at 25, 50, 100, and 200 mg/kg. Significant difference: ^∗∗^*P* < 0.01 compared with NC.

**Table 4:** Effect of the hydroethanolic extract of *Bixa orellana* leaves on the mass of the small intestine and its contents in mice.

Treatment (mg/kg)	Mass of small intestine with content (g)	Mass of empty small intestine (g)	Mass of small intestinal content (g)
NC	1.13 ± 0.02	0.92 ± 0.09	0.21±0.01^∗∗^
DC	1.38 ± 0.19	1.01 ± 0.09	0.37 ± 0.13
LOP5	1.07 ± 0.03	0.81 ± 0.07	0.26±0.06^∗∗^
HEBo50	1.23 ± 0.08	0.90 ± 0.09	0.33 ± 0.04^∗^^b^
HEBo100	1.43 ± 0.11	1.15 ± 0.08	0.28±0.07^∗∗^^b^
HEBo200	1.38 ± 0.07	1.14 ± 0.09	0.24±0.09^∗∗^^a^

Values are expressed as mean ± S.E.M (*n* = 5). NC: normal control; DC: diarrheal control; Lop5: Loperamide 5 mg/kg; HEBo50, HEBo100, and HEBo200: hydroethanolic extract of *Bixa orellana* leaves, respectively, at 50, 100, and 200 mg/kg. Significant difference: ^∗^*P* < 0.05 and ^∗∗^*P* < 0.01 compared with DC; ^a^*P* < 0.05 and ^b^*P* < 0.01 compared with NC.

**Table 5:** Effect of hydroethanolic extract of *Bixa orellana* leaves on electrolyte concentration in intestine content in mice.

Groups	[Cl^−^] (mEq/L)	[Na^+^] (mEq/L)	[K^+^] (mEq/L)
NC	97.59±7.08^∗∗^	102.84±4.22^∗∗^	10.51 ± 2.74^∗^
DC	126.39 ± 4.18	129.60 ± 8.24	5.75 ± 1.01
Lop5	91.27±8.17^∗∗^	103.16±3.82^∗∗^	11.05 ± 3.41^∗^
HEBo50	103.18±6.14^∗∗^	107.82±4.46^∗∗^	9.89 ± 0.68^∗^
HEBo100	108.61±5.12^∗∗^	106.91±6.47^∗∗^	10.54±0.43^∗∗^
HEBo200	106.39±3.08^∗∗^	106.33±4.85^∗∗^	10.02 ± 2.91^∗^

Data are expressed as mean ± S.E.M (*n* = 5). [Cl^−^]: chloride ion concentration; [Na^+^]: sodium ion concentration; [K^+^]: potassium ion concentration; NC: normal control; DC: diarrheal control; Lop5: Loperamide 5 mg/kg; HEBo50, HEBo100, and HEBo200: hydroethanolic extract of *Bixa orellana* leaves, respectively, at 50, 100, and 200 mg/kg. Significant difference: ^∗^*P* < 0.05 and ^∗∗^*P* < 0.01 compared with DC.

## Data Availability

The data used to support the findings of this study are available from the corresponding author upon request.
